# Blood lead level in infants and subsequent risk of malaria: A prospective cohort study in Benin, Sub-Saharan Africa

**DOI:** 10.1371/journal.pone.0220023

**Published:** 2019-07-18

**Authors:** Amanda Garrison, Babak Khoshnood, David Courtin, Jacqueline Milet, André Garcia, Achille Massougbodji, Pierre Ayotte, Michel Cot, Florence Bodeau-Livinec

**Affiliations:** 1 INSERM UMR1153 Equipe de recherche en Epidémiologie Obstétricale, Périnatale, et Pédiatrique (EPOPé), Center for Epidemiology and Statistics, Sorbonne Paris Cité (CRESS), Paris, France; 2 Sorbonne Universités, UPMC Université Paris 6, Paris, France; 3 Ecole des Hautes Etudes en Santé Publique (EHESP), Saint Denis, France; 4 Mère et enfant face aux infections tropicales (MERIT), l’Institut de Recherche pour le Développement (IRD), Université Paris 5, Sorbonne Paris Cité, Paris, France; 5 Institut de Recherche Clinique du Bénin, Abomey-Calavi, Bénin; 6 Institut National de Santé Publique du Québec, Québec City, Canada; Instituto Rene Rachou, BRAZIL

## Abstract

Lead and malaria both present significant health risks to children in Sub-Saharan Africa. Previous studies have shown that high blood lead levels in children act as a protective factor against subsequent malaria incidence. The main objective of this study was to investigate associations between blood lead level and malaria outcomes prospectively in Beninese children from 12 to 24 months of age. Two-hundred and four children were assessed for lead at 12 months and closely followed until 24 months for malaria; when symptoms and parasite density were also recorded. Univariate and multivariate negative binomial and linear regression models tested associations between blood lead level quartile and total episodes of malaria (total symptomatic and asymptomatic episodes) and parasite density, respectively. Median blood lead level among children measured at 12 months was 56.50 (4.81–578) μg/L. During the 12-month follow-up, 172 (84.31%) children had at least one malaria episode. Univariate and multivariate negative binomial and linear regressions did not reveal significant associations between blood lead level quartile and malaria outcomes. Iron deficiency was not found to be an effect modifier. Results from this prospective child-cohort study investigating associations between blood lead level and malaria did not confirm results from previous cross-sectional studies. Further research is needed to further explore this relationship and other co-morbidities due to malaria and lead.

## Introduction

Lead (Pb), a toxic heavy metal with a blood half-life time between 30–40 days [1][2], is found in gasoline, paint, contaminated soil, ammunition, and water pipes [3] and can have permanent, harmful effects on the human body if ingested or inhaled [4]. Elevated levels of lead exposure (defined by the Centers for Disease Control (CDC) as a blood lead level of 50 μg/L or more [5]) have specific consequences on child health, including decreased Intelligence Quotient (IQ), decreased attention span, and increased antisocial behaviors [6][7][8]. Impacts of lead on child health and development are particularly pertinent in developing countries, such as Benin, where exposure to lead is still relatively high. A cohort study involving children and their mothers in Benin, Sub-Saharan Africa in 2016 identified high levels of lead exposure in the population, with 58% of children having blood lead levels greater than 50μg/L [9].

Malaria also presents significant health risks for children in Benin, where malaria caused primarily by the *Plasmodium falciparum* parasite is the leading cause of death in children under five years of age in the country and the main cause of morbidity in adults [10]. The incidence rate of malaria in Benin is 293 cases per 1,000 population at risk, with 100% of the population considered at risk of developing malaria [11]. In areas of the world with high transmission of malaria, exposed children develop an acquired clinical immunity after repeated infections and tend to exhibit more asymptomatic than symptomatic malaria episodes over time [12]. With children at higher risk of death from malaria in Benin, it is important to identify specific risk factors and potential co-exposures present in their environments, such as lead and other heavy metals that may impact their risk of disease.

Thus far, only cross-sectional investigations of associations between blood lead level and malaria have been conducted in child populations, one involving 653 Nigerian children ages 2 to 9 years [13] and one involving 203 children in Benin at 12 months of age [14]. Both studies observed significant negative associations between high blood lead levels and malaria risk in children. Based on these previous studies, we identified the need for a prospective analysis of associations between lead and malaria, while taking into consideration iron deficiency as a potential effect modifier. We also sought to investigate symptomatic and asymptomatic malaria episodes and parasite density in children as secondary outcomes.

The aim of our study was to analyze data in a cohort of Beninese children followed between the ages of 12 to 24 months in order to study the associations between blood lead levels taken between 10 to 13 months of age and subsequent malaria episodes up to 24 months of age. We hypothesized that children with high blood lead level (in higher blood lead level quartiles) at 12 months of age would be associated with fewer number of subsequent malaria episodes and lower parasite density between ages 12 to 24 months in Benin compared to children within the lowest blood lead level quartile.

## Methods

### Study population

This was an observational, prospective cohort study following children born to women enrolled in a randomized clinical trial (Malaria in Pregnancy Preventive Alternative Drugs, MiPPAD, NCT00811421) investigating two intervention therapies for malaria during pregnancy in the semi-rural area of Allada, 40 kilometers north of Cotonou, in Benin, Sub-Saharan Africa. Study protocol and inclusion criteria of participants for the clinical trial is explained elsewhere [15]. All singleton children born to mothers from the MiPPAD trial were invited to participate in a subsequent cross-sectional study (TOVI) at 12 months of age [16]. A sub-cohort of these children was then followed closely to measure malaria incidence and parasite density between 12 to 24 months of age within the TOLIMMUNPAL study. Participating children in our analyses included those who met the following inclusion criteria: had mothers who met the original inclusion criteria for the MiPPAD clinical trial, were assessed for blood lead level within the TOVI study between 10 to 13 months of age, and were followed for malaria incidence within the TOLIMMUNPAL study from 12 to 24 months of age.

### Exposure of interest

Blood lead level, the primary exposure of interest in this study, was measured once in children ages 10–13 months within the TOVI study. Eight mL of venous blood was collected from each child and 4mL put into a tube with dipotassium EDTA. An aliquot of EDTA blood was diluted 20-fold in ammonia 0.5% v/v and 0.1% v/v surfactant Triton-X and analyzed by inductively coupled plasma mass spectrometry (ICP-MS; Perkin Elmer Sciex Elan DRC II ICP-MS instrument) at the Centre de Toxicologie, Institut National de Santé Publique du Québec (Québec, Canada). The limit of detection for blood lead analysis was 0.2 μg/L [9]. Blood lead level was analyzed in quartiles due to the non-linear relationship between lead and malaria and in order to better interpret results.

### Outcomes of interest

There were two primary outcomes of interest within this study, malaria and parasite density in children. Malaria was diagnosed in children from 12 to 24 months of age through a positive thick blood smear test and/or rapid diagnostic test (RDT) within the TOLIMMUNPAL study. A positive thick blood smear test, the gold standard of malaria diagnosis, involves the examination of blood samples using microscopy in order to diagnosis the presence of parasites [17,18]. Pan/Pf RDT (Parascreen), able to give results within 15 minutes of administration, identifies the presence of *Plasmodium*-specific histidine-rich protein-2 produced in the infected human blood [19]. Each month during the 12-month follow-up, participating children were visited by a nurse, who performed a thick blood smear test to diagnose malaria, regardless of symptoms, and an RDT if they presented with fever (temperature equal to or greater than 37.5°C). A nurse also visited children at home every 15 days, during which time a thick blood smear test and an RDT were administered if children had fever or history of fever in the previous 24 hours. Additionally, children and their families had access to free emergency care and were able to attend the clinic if symptoms occurred. During these visits, a thick blood smear test and an RDT were administered. Symptomatic malaria was defined as a positive thick blood smear and/or RDT and the presence of fever within three days of diagnosis. Parasite density (in parasites/μL) was measured in children positively diagnosed with malaria by a thick blood smear test, through the use of a multiplication factor applied to the average parasitemia/field [14].

Malaria was analyzed as the total number of malaria episodes, total number of symptomatic episodes, and total number of asymptomatic episodes during the 12-month follow-up. Parasite density was log-transformed and analyzed as mean logarithm parasite density per child.

### Potential confounders

Potential confounders included in adjusted models were socioeconomic status (SES), maternal education, iron deficiency, mosquito-net use, malaria status before 12 months, maternity ward location, and environmental risk of infection. Information regarding maternal education, mosquito-net use, and maternity ward were collected via questionnaire given to mothers during follow-up. Serum ferritin concentrations were measured in children using an AxSym Immuno-Assay Analyzer (Abbott Laboratories, Abbott Park, IL) with a sample of 500 mL of serum. Iron deficiency was defined as a serum ferritin concentration less than 12 μg/L or as serum ferritin concentration of 12 to 70 μg/L in the presence of inflammation (CRP concentration > 5mg/L) [20]. In addition to being considered as a potential confounding factor, iron deficiency was also tested as an effect modifier. Malaria status before 12 months was defined as having at least one malaria episode before the lead assessment at 12 months of age.

TOLIMMUNPAL researchers collected information on mosquito density, as well as environmental (rainfall, soil type, nearby water sources, vegetation index) and biological data (number of inhabitants per room in household, use of bed nets and/or insecticides) in order to calculate a time- and space-dependent environmental risk of infection quantifying each child’s exposure to malaria vectors using a predictive model [21].

### Statistical analysis

Univariate analyses of associations between the primary exposure of interest, blood lead level, the primary outcomes of interest, total malaria episodes and mean logarithm parasite density, and potential confounders were performed. Confounders found to be associated to the primary exposure and/or outcomes (p<0.20) were kept in final, adjusted models. Multivariate negative binomial regression models were run to examine associations between blood lead level and number of malaria episodes, including number of symptomatic and asymptomatic malaria episodes separately. Linear regression models tested associations between blood lead level and mean logarithm parasite density measured in children.

Additionally, characteristics of children lost to follow-up (i.e. not present at 24 months) were compared to those present for the entire follow-up using t-tests, Wilcoxon-rank sum tests, and Fisher exact tests when appropriate. Two sensitivity analyses were conducted: (1) regression analyses after removal of children potentially exposed to lead through contaminated paint chips, and (2) regression analyses including only malaria episodes diagnosed within first 6 months after lead assessment, due to the 30-day half life of lead. Statistical analyses were completed using STATA 14.2 (StataCorp. 2015. *Stata Statistical Software*: *Release 14*. College Station, TX: StataCorp LP) for Windows.

### Ethical consideration

All studies from which data were used for the purpose of this study were approved by the following bodies: the Hospital Clinic of Barcelona (Spain), the Comité Consultatif de Déontologie et d’Éthique of the Institut de Recherche pour le Développement (France) [15], the University of Abomey-Calavi in Benin and New York University in the United States (IRB#09–1253) [9], and the Beninese Ethical Committee of the Faculté des Sciences de la Santé (FSS).

## Results

**Fig 1:**
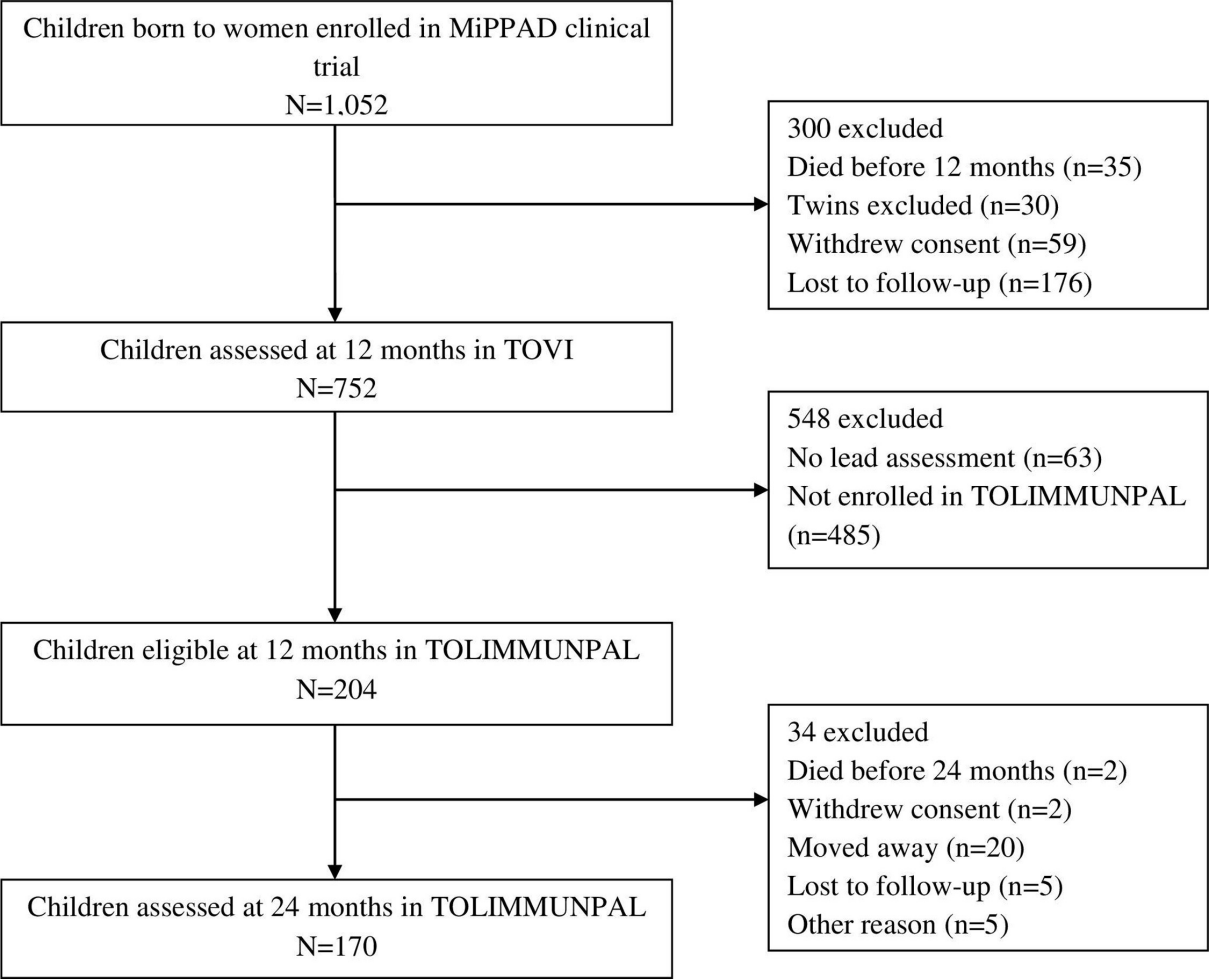
Population follow-up. Follow-up of participating children from birth to 24 months of age.

The follow-up of children prior to and during the TOLIMMUNPAL study is illustrated in Fig 1. Of the 204 children eligible for our study for which data was available on lead assessment at 12 months of age and malaria follow-up, 170 (83.33%) were present at 24 months of age. All regression analyses included all children and took into account the differing follow-up times of children during the 12-month period.

Demographic and clinical characteristics of children are shown in Table 1. Median blood lead level among children was 56.50 (±56.83) μg/L. During follow-up, 172 (84.31%) children had at least one malaria episode, with 40 (19.61%) having one episode, 41 (20.10%) having two episodes, and 91 (44.61%) having more than two malaria episodes during the 12 months. Of all occurring malaria episodes, 69.45% of them were symptomatic episodes. Median parasite density per child was 1,674.40 (±6,396.53) parasites/μL.

**Table 1:** Demographic and clinical characteristics of participating children (N = 204).

Parameter	Category	Mean (±SD)^1^ or n (%)
Sex		204 (100%)
	Female	104 (50.98%)
	Male	100 (49.02%)
Age at lead assessment (months)		11.98 (±0.36)
Blood lead level (μg/L)		56.50 (±56.83)
Ferritin concentration (μg/L)		20.15 (±98.73)
Inflammation (CRP>5mg/L)		204 (100%)
	Yes	99 (48.53%)
	No	105 (51.47%)
Iron deficiency (ferritin<12 μg/L or 12–70 μg/L if CRP>5mg/L)		198 (100%)
	Yes	95 (47.98%)
	No	103 (52.02%)
Socioeconomic status		200 (100%)
	Lowest	88 (44%)
	Medium	67 (33.50%)
	Highest	45 (22.50%)
Maternal education		200 (100%)
	Some	86 (42.80%)
	None	115 (57.20%)
Maternity ward location		201 (100%)
	Attogon	51 (25.37%)
	Sékou	150 (74.63%)
Use of mosquito nets in house		189 (100%)
	Rare	4 (2.12%)
	Occasional	15 (7.94%)
	Frequent	24 (12.70%)
	Always	146 (77.25%)
Environmental risk		0.04 (±0.17)
Malaria status before 12 months		204 (100%)
	No malaria before 12 months	152 (74.51%)
	At least one malaria episode before 12 months	52 (25.49%)
Malaria status of children during follow-up		204 (100%)
	No malaria	32 (15.69%)
	One episode	40 (19.61%)
	Two episodes	41 (20.10%)
	More than two episodes	91 (44.61%)
Total malaria episodes		586 (100%)
	Symptomatic	407 (69.45%)
	Asymptomatic	179 (30.55%)
Parasite density per child (parasites/μL)		1,674 (±6,396)

^1^Median (±SD) shown for variables with skewed distribution.

**Fig 2:**
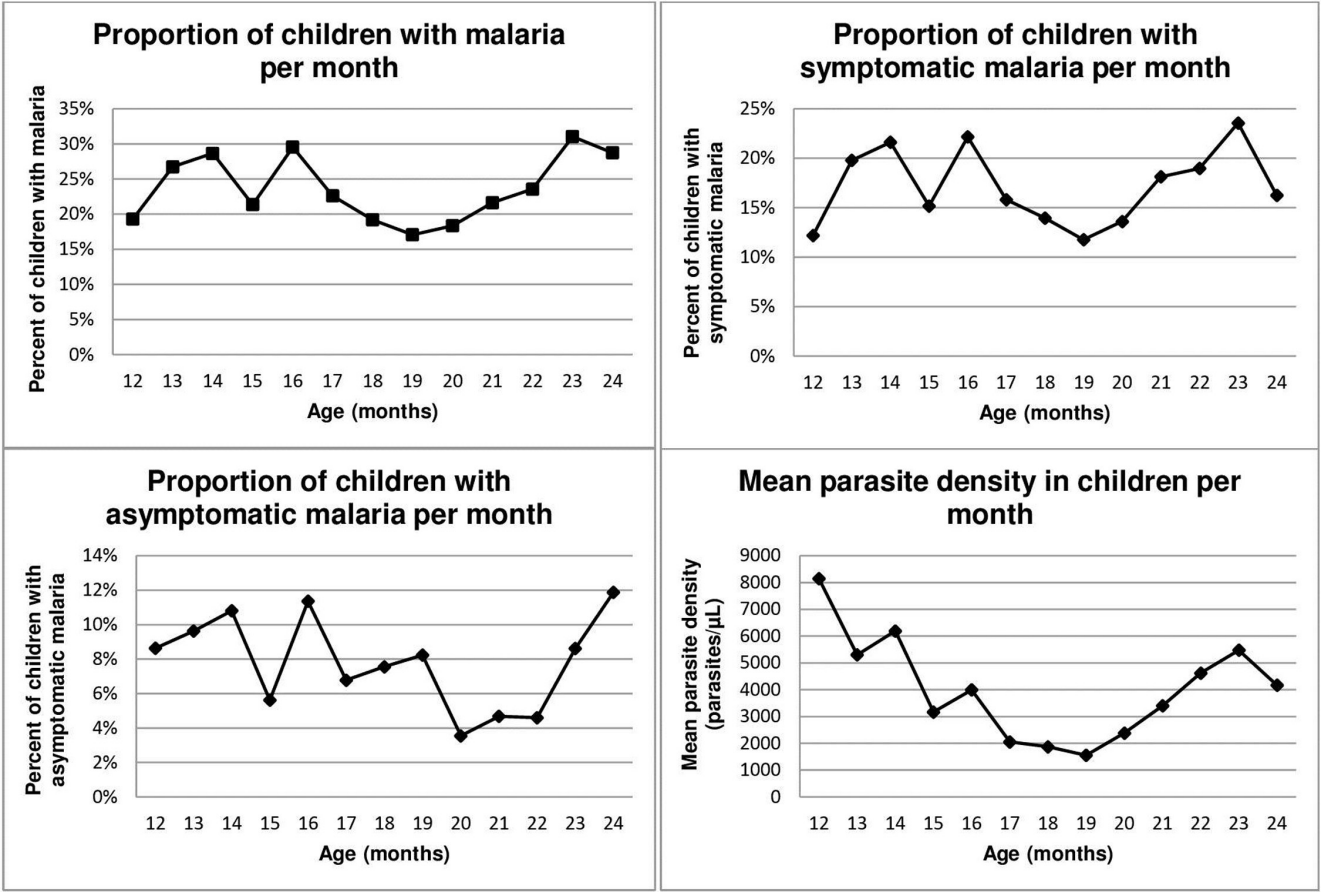
Proportion of children diagnosed with malaria and parasite densities each month of follow-up.

Trends in malaria incidence and prevalence and parasite density were examined in monthly intervals during the follow-up period to further describe our population (Fig 2). At 12 months of age, 19% of children were positively diagnosed with malaria, 12% had symptomatic malaria and 9% had asymptomatic malaria. After 12 months, malaria incidence decreased slightly each month until 20 months of age when incidence increased. By the end of the study period, roughly 29% of children were diagnosed with malaria; with 16% having symptomatic malaria and 12% having asymptomatic malaria. Mean parasite density in children was highest at 12 months (8,148 parasites/μL), although malaria prevalence was relatively low at this age.

Univariate analyses were carried out between potential confounders and outcome variables (total malaria episodes and parasite density) and kept in final models if found significant (p<0.20) (Tables 2 and 3). No significant associations were found in univariate analyses between blood lead level quartile and any malaria outcomes. Children born to mothers with a primary education were found to have lower incidence rate ratio of total symptomatic episodes [0.81 (0.65, 0.99)] and lower mean logarithm parasite density [-0.15 (-0.30, -0.01)] compared to children born to mothers without at least a primary education. Increased environmental risk was also found to be significantly associated to higher incidence rate ratios for total malaria episodes [2.89 (1.44, 5.80)], total symptomatic episodes [1.95 (1.07, 3.54)], and higher mean logarithm parasite density [0.53 (0.09, 0.97)]. Children examined at the maternity ward in Sékou were also found to have significantly higher incidence rate ratios of total malaria episodes [1.36 (1.03, 1.81)], total symptomatic episodes [1.37 (1.05, 1.78)], and higher mean logarithm parasite density [0.17 (0.01, 0.34)]. Neither socioeconomic status nor iron deficiency were found to be significantly associated to either outcome, but were kept in final models due to their previously reported associations with malaria. Iron deficiency was also found to be strongly positively associated with lead (p<0.01) in univariate analyses.

**Table 2:** Univariate negative binomial regression between potential confounders and malaria outcomes^†^.

Parameter	Category	Total malaria episodes IRR^1^ (95% CI)	P-value	Total symptomatic episodes IRR (95% CI)	P-value	Total asymptomatic episodes IRR (95% CI)	P-value
Blood lead level quartile			0.80		0.62		0.86
	1^st^ [Ref]	1		1		1	
	2^nd^	0.89 (0.65, 1.24)		1.00 (0.75, 1.33)		0.85 (0.42, 1.70)	
	3^rd^	0.99 (0.71, 1.36)		1.06 (0.80, 1.41)		0.80 (0.40, 1.59)	
	4^th^	0.87 (0.62, 1.22)		0.88 (0.66, 1.19)		0.69 (0.34, 1.41)	
Iron deficient			0.80		0.99		0.41
	No [Ref]	1		1		1	
	Yes	1.03 (0.82, 1.30)		0.99 (0.81, 1.22)		1.23 (0.75, 2.03)	
Maternal education			0.09		<0.01		0.71
	None [Ref]	1		1		1	
	Some	0.81 (0.64, 1.03)		0.81 (0.65, 0.99)		1.21 (0.74, 2.00)	
Socioeconomic status quartile			0.47		0.71		0.48
	1^st^ [Ref]	1		1		1	
	2^nd^	0.89 (0.66, 1.19)		0.94 (0.72, 1.22)		0.80 (0.43, 1.49)	
	3^rd^	0.76 (0.54, 1.11)		0.86 (0.62, 1.19)		0.69 (0.32, 1.50)	
	4^th^	0.81 (0.58, 1.12)		1.00 (0.75, 1.33)		0.63 (0.31, 1.29)	
Mosquito Net Use			0.30		0.25		0.39
	Rarely [Ref]	1		1		1	
	Occasionally	0.59 (0.27, 1.29)		0.88 (0.46, 1.71)		0.22 (0.04, 1.18)	
	Frequently	0.66 (0.31, 1.42)		0.82 (0.43, 1.56)		0.41 (0.09, 1.94)	
	Always	0.55 (0.28, 1.10)		0.76 (0.42, 1.36)		0.44 (0.11, 1.80)	
Malaria before 12 months			0.56		0.50		0.64
	No [Ref]	1		1		1	
	Yes	1.08 (0.83, 1.40)		0.98 (0.78, 1.23)		1.03 (0.59, 1.79)	
Environmental risk		2.89 (1.44, 5.80)	<0.01	1.95 (1.07, 3.54)	<0.01	2.78 (0.58, 13.36)	0.13
Maternity ward			0.03		<0.01		0.79
	Attogon [Ref]	1		1		1	
	Sékou	1.36 (1.03, 1.81)		1.37 (1.05, 1.78)		1.02 (0.56, 1.85)	

^†^Sample sizes for children differ among characteristics due to missing values.

^1^IRR = Incidence Rate Ratio

**Table 3:** Univariate linear regression between potential confounders and mean logarithm parasite density^†^.

Parameter	Category	Parasite density (mean logarithm) Coefficient (95% CI)	P-value
Blood lead level quartile			0.25
	1^st^ [Ref]	0	
	2^nd^	-0.19 (-0.39, 0.02)	
	3^rd^	-0.16 (-0.37, 0.05)	
	4^th^	-0.18 (-0.39, 0.03)	
Iron deficient			0.45
	No [Ref]	0	
	Yes	-0.06 (-0.20, 0.09)	
Maternal education			0.04
	None [Ref]	0	
	Some	-0.15 (-0.30, -0.01)	
Socioeconomic status quartile			0.23
	1^st^ [Ref]	0	
	2^nd^	-0.08 (-0.27, 1.11)	
	3^rd^	-0.20 (-0.42, 0.02)	
	4^th^	-0.17 (-0.38, 0.03)	
Mosquito Net Use			0.15
	Rarely [Ref]	0	
	Occasionally	-0.32 (-0.76, 0.11)	
	Frequently	-0.14 (-0.55, 0.28)	
	Always	-0.29 (-0.68, 0.10)	
Malaria before 12 months			0.15
	No [Ref]	0	
	Yes	0.12 (-0.04, 0.29)	
Environmental risk		0.53 (0.09, 0.97)	0.02
Maternity ward			0.05
	Attogon [Ref]	0	
	Sékou	0.17 (0.01, 0.34)	

^†^Sample sizes for children differ among characteristics due to missing values.

Multivariate negative binomial regression models were carried out between blood lead level quartile and total number of malaria episodes, total number of symptomatic episodes, and total number of asymptomatic episodes while adjusting for potential confounding factors (Table 4). Higher blood lead level quartiles were not found to be significantly associated with any outcome in multivariate analyses, as hypothesized, although children in the 4^th^ (highest) blood lead level quartile had the lowest incidence rate ratios for total malaria episodes [0.94 (0.68, 1.30)], total symptomatic episodes [0.92 (0.68, 1.24)], and total asymptomatic episodes [0.62 (0.29, 1.31)] compared to children in the 1^st^ (lowest) quartile.

**Table 4:** Multivariate negative binomial regression models for associations between blood lead level quartile and malaria outcomes^†^.

Blood lead level quartile	Total malaria episodes (N = 183) IRR (95% CI)	P-value	Total symptomatic episodes (N = 166) IRR (95% CI)	P-value	Total asymptomatic episodes (N = 166) IRR (95% CI)	P-value
1^st^ [Ref]	1		1		1	
2^nd^	1.00 (0.73, 1.36)	1.00	1.08 (0.81, 1.43)	0.60	0.78 (0.38, 1.57)	0.51
3^rd^	1.02 (0.74, 1.40)	0.92	1.06 (0.79, 1.40)	0.42	0.78 (0.38, 1.63)	0.68
4^th^	0.94 (0.68, 1.30)	0.70	0.92 (0.68, 1.24)	0.82	0.62 (0.29, 1.31)	0.29

†Adjusted for iron deficiency, maternal education, socioeconomic status, mosquito net use, environmental risk, and maternity ward location

Multivariate linear regression analyses between blood lead level quartile and mean logarithm parasite density found reduced coefficient estimates for parasite density in children within the 2^nd^ [-0.02 (-0.18, 0.13)], 3^rd^ [-0.03 (-0.19, 0.13)], and 4^th^ [-0.04 (-0.20, 0.12)] quartiles, however no associations were found to be statistically significant (Table 5).

**Table 5:** Multivariate linear regression for associations between blood lead level quartile and mean logarithm parasite density^†^.

Blood lead level quartile	Parasite density (mean logarithm) (N = 183) Coefficient (95% CI)	P-value
1^st^ [Ref]	0	
2^nd^	-0.02 (-0.18, 0.13)	0.76
3^rd^	-0.03 (-0.19, 0.13)	0.69
4^th^	-0.04 (-0.20, 0.12)	0.65

†Adjusted for iron deficiency, maternal education, socioeconomic status, mosquito net use, environmental risk, malaria status before 12 months, and maternity ward location.

Secondary analyses were stratified by iron deficiency and an interaction term for iron deficiency was tested for significance to reveal potential effect modification; however, no significant effect modification was identified and the interaction term was not kept in final, multivariate models.

Sensitivity analysis removing children with potential acute post-natal exposure to lead through ingestion of fallen paint chips (n = 23) did not reveal significantly different associations between blood lead level quartile and malaria outcomes in negative binomial and linear regression models (S2 Table). Sensitivity analyses including only malaria episodes that occurred in the 6 months followed lead assessment (ages 12–18 months) were conducted (S3 Table). While these analyses revealed lower effect estimates in associations between blood lead level and malaria outcomes, results were not statistically different from analyses including all malaria outcomes from the 12-month study period.

## Discussion

Children in higher blood lead level quartiles were not found to have significantly lower rates of malaria or lower parasite densities compared to children in the lowest blood lead level quartile, as hypothesized. Our prospective findings do not support the cross-sectional analyses by Moya-Alvarez et al (2016) which found significant negative associations between elevated blood lead level and malaria incidence and parasite density at 12 months of age in Beninese children.

The ingestion of lead-based paint chips by children is an acute post-natal exposure pathway that varies with age when children begin to exhibit hand-to-mouth behaviors and are more likely to pick up contaminated paint chips off the ground in their homes and ingest them [22]. For this reason, sensitivity analyses were conducted excluding these children exposed to lead via paint chips (n = 23), as this type of exposure could have led to higher and more variable blood lead levels during the 12-month follow-up, thus potentially under-estimating the level of lead exposure after 12 months in our population. However, these sensitivity analyses did not reveal different results when excluding these children.

Limitations of this study include the small sample size, missing information for certain confounding factors which resulted in the loss of these children in analyses, and the fact that blood lead level was measured only once in children at the onset of the study period. Fluctuations in blood lead levels could have occurred, however since they were not measured at multiple intervals during the 12-month study period, it is difficult to ascertain their exact fluctuations and effects on malaria outcomes over time. The half-life of lead in blood is estimated between 30–40 days, although lead accumulated in bone may have a half-life of several decades [2] which can act as a source of lead transfer from mother to fetus during pregnancy [23]. Sensitivity analyses were conducted to include only malaria episodes and parasite densities measured in the first 6 months after lead assessment to account for blood-lead half-life; however these analyses did not reveal significantly different results from analyses including all malaria outcomes during the entire follow-up. Demographic characteristics of children present and absent at 24 months of age were compared to assess any potential differences in these populations (S1 Table). Median blood lead level was significantly higher in children lost to follow-up (68.55 μg/L [17.00–260.00]) compared to those not lost (45.20 μg/L [36.00–73.4]). Use of mosquito nets in households was also significantly different between children present and absent at the final visit (p<0.04), with children absent at the 24-month visit less likely to have always used mosquito nets during the follow-up, compared to children present at the final visit. Therefore, children lost to follow-up were more exposed and more at risk of developing malaria, meaning that analyses within this paper potentially underestimate associations between blood lead level and subsequent malaria episodes in our population.

Based on the strong negative associations found between blood lead level and malaria, including parasite density, in this population of children cross-sectionally at 12 months of age [14], we expected to see similar results over a prospective period of time. However, lead no longer seemed to have a significant protective impact on malaria incidence after 12 months of age in children. Numerous studies within malaria-endemic regions of the world have exhibited identified iron deficiency as a protective factor against malaria [24–26]. However, we found no significant associations between iron deficiency status in children and malaria outcomes in univariate analyses to support these previous findings. These results could be due to the nature of the prospective follow-up of this study, during which time children received iron supplements to treat iron-deficiency anemia. Iron deficiency was only assessed at 12 months of age. In total, follow-up in the study may have resulted in lowering iron deficiency and malaria as explained earlier between 12 and 24 months of age.

The mechanism between blood lead level and malaria is not well known, and currently very little literature exists on this topic. Nriagu et al proposed three biologically plausible potential mechanisms to explain their observed negative associations between blood lead level and malaria in children (2008). Lead, which is absorbed and stored within red blood cells (RBCs), could inhibit the life cycle of the *Plasmodium* parasite which lives and replicates within RBCs as well. Lead poisoning has been shown to inhibit protein synthesis and certain cellular processes [27,28], and its accumulation in RBCs could lead to poor utilization of iron by *Plasmodia* during development [29]. Another hypothesis is that the phenotype-specific immune response to lead poisoning in the body, which leads to the production of T-helper 2 (Th2) cells, may contribute to a T-helper 1 (Th1)/Th2 balance and thus promote protection against malaria [30,31]. Iron deficiency could also play a role in the mechanism between lead and malaria, as studies have shown iron deficiency to be associated with increased lead uptake [29] and lower risk of malaria in high-transmission areas [25,32]. Our analyses attempted to explore this potential mechanism by testing iron deficiency as an effect modifier in analyses, however iron deficiency was not found to be significant and thus the association was not further investigated.

Between 6–12 months of age, children are no longer protected by immunity passed on to them through their mothers’ antibodies and they begin experiencing their first malaria episodes. Malaria incidence fluctuated from month to month during the follow-up period as children were infected with malaria and were subsequently treated within the confines of the study. Of interest is the observation that mean parasite density in children was highest at 12 months, although malaria prevalence was relatively low at this point (18%). As children were more actively screened at and after 12 months and treated for malaria if positively diagnosed, *Plasmodium* concentrations generally declined in children after 12 months. Subsequently, the proportion of children with malaria decreased. This may be due to a large proportion of children who were treated after active screening around 12 months of age. Towards the end of the study period, the incidence of asymptomatic malaria episodes increased and the incidence of symptomatic episodes decreased between 23–24 months of age, which reflects the acquired immunity typically observed in children born into high malaria transmission settings. Therefore, malaria between 12 and 24 months was highly affected by the nature of the follow-up for this study, which potentially biases results found within our analyses towards the null. This may explain why this present study with a longitudinal follow-up does not confirm results found at 12 months of age.

The high blood lead levels measured within this population of infants make a case for the need for more efficient lead exposure identification and control in Benin. Iron deficiency leads to increased lead absorption, as indicated in the literature and in our study, further emphasizing the need for effective strategies to prevent and treat iron deficiency in vulnerable populations. Many populations in Sub-Saharan Africa are exposed to several co-morbidities, such as anemia, lead poisoning, malaria, helminthes, and malnutrition. These co-morbidities should be more closely evaluated and their impacts on one another investigated in order to estimate the true burden of disease in these nations and improve existing prevention and treatment strategies.

## Conclusions

In conclusion, our prospective study does not confirm previous cross-sectional findings on associations between blood lead level and malaria in children. Iron deficiency is shown to be strongly positively correlated to increased lead absorption, suggesting that prevention and treatment of iron deficiency should be in accordance with strategies to reduce lead exposure in vulnerable populations. Malaria prevalence remains high in Benin, with the highest mortality rates in children under 5 years of age. Due to the numerous co-morbidities faced by populations in developing countries, further research should investigate interactions between these co-morbidities and further explore the existing mechanisms between lead and malaria in order to improve prevention and treatment strategies.

## Supporting information

S1 Table Demographic characteristics of children present and absent at the final systematic visit at 24 months of age. (DOCX)

S2 Table (A) Multivariate negative binomial regression results excluding children potentially exposed to lead by ingestion of fallen paint chips. (B) Multivariate linear regression results excluding children potentially exposed to lead by ingestion of fallen paint chips.
(DOCX)

S3 Table (A) Multivariate negative binomial regression results for first 6 months after lead assessment. (B) Multivariate linear regression results for first 6 months after lead assessment.
(DOCX)

S1 File TOVI database used in analyses. (PDF)

S2 File TOLIMMUNPAL database used in analyses. (PDF)
