# Supplementary material for: Blood lead level in infants and subsequent risk of malaria: A prospective cohort study in Benin, Sub-Saharan Africa
Source: PLoS One. 2019 Jul 18;14(7):e0220023. doi: 10.1371/journal.pone.0220023 (PMC6638975; doi:10.1371/journal.pone.0220023)
Supplement: S1 Table — (DOCX) [file pone.0220023.s001.docx]

**S1 Table. Demographic characteristics of children present and absent at the final systematic visit at 24 months of age.**

| Variable | Category | Present at 24 months (n=170)^1^ | Absent at 24 months (n=34)^1^ | P-value* |
| --- | --- | --- | --- | --- |
| Sex |  |  |  | 0.61 |
|  | Female | 85 (50.30%) | 15 (45.45%) |  |
|  | Male | 84 (49.70%) | 18 (54.55%) |  |
| Age at lead assessment (months) |  | 11.99 (+0.36) | 11.99 (+0.35) | 0.99 |
| Blood lead level (µg/L)^2^ |  | 45.20 (36.00-73.4) | 68.55 (17.00-260.00) | 0.03 |
| Ferritin concentration (µg/L)^2^ |  | 35.45 (9.70-232.60) | 20.70 (0.40-261.70) | 0.91 |
| Inflammation (CRP>5mg/L) |  |  |  | 0.35 |
|  | Yes | 80 (47.06%) | 19 (55.88%) |  |
|  | No | 90 (52.94%) | 15 (44.12%) |  |
| Iron deficiency |  |  |  | 0.73 |
|  | Yes | 81 (48.50%) | 14 (45.16%) |  |
|  | No | 86 (51.50%) | 17 (54.85%) |  |
| Socioeconomic status |  |  |  | 0.31 |
|  | Lowest | 71 (42.26%) | 17 (53.13%) |  |
|  | Medium | 60 (35.71%) | 7 (21.88%) |  |
|  | Highest | 37 (22.02%) | 8 (25.00%) |  |
| Maternal education |  |  |  | 0.79 |
|  | Primary education | 73 (43.20%) | 13 (32.00%) |  |
|  | No primary education | 96 (56.80%) | 19 (59.38%) |  |
| Maternity ward location |  |  |  |  |
|  | Attogon | 40 (23.81%) | 11 (33.33%) | 0.25 |
|  | Sékou | 128 (76.19%) | 22 (67.67%) |  |
| Use of mosquito nets in house |  |  |  | 0.02 |
|  | Rare | 4 (2.35%) | 0 (0%) |  |
|  | Occasional | 14 (8.24%) | 1 (5.26%) |  |
|  | Frequent | 17 (10.00%) | 8 (36.84%) |  |
|  | Always | 135 (79.41%) | 11 (57.89%) |  |
| Malaria status before 12 months |  |  |  | 0.78 |
|  | No malaria | 126 (74.12%) | 26 (76.47%) |  |
|  | At least one malaria episode | 44 (25.88%) | 8 (23.53%) |  |

^*^P-value results from Fisher exact tests and Student t-tests with a significance of P<0.05.

^1^Sample sizes for children differ among characteristics due to missing values.

^2^Median (range) shown for variables with skewed distributions.
