# Supplementary material for: Blood lead level in infants and subsequent risk of malaria: A prospective cohort study in Benin, Sub-Saharan Africa
Source: PLoS One. 2019 Jul 18;14(7):e0220023. doi: 10.1371/journal.pone.0220023 (PMC6638975; doi:10.1371/journal.pone.0220023)
Supplement: S2 Table — (A) Multivariate negative binomial regression results excluding children potentially exposed to lead by ingestion of fallen paint chips. (B) Multivariate linear regression results excluding children potentially exposed to lead by ingestion of fallen paint chips. (DOCX) [file pone.0220023.s002.docx]

**Table A in S2 Table. Multivariate negative binomial regression results excluding children potentially exposed to lead by ingestion of fallen paint chips^§^.**

| Factor | Total malaria episodes (N=162)* | Total symptomatic episodes (N=162)* | Total asymptomatic episodes (N=162)* |
| --- | --- | --- | --- |
| Blood lead level quartile |  |  |  |
| 1^st^ | 1 | 1 | 1 |
| 2^nd^ | 1.01 (0.74, 1.39) | 1.09 (0.80, 1.47) | 0.83 (0.40, 1.74) |
| 3^rd^ | 1.03 (0.74, 1.43) | 1.16 (0.85, 1.57) | 0.88 (0.39, 1.96) |
| 4^th^ | 0.98 (0.70, 1.38) | 0.96 (0.69, 1.34) | 0.84 (0.39, 1.86) |

^§^ Incidence rate ratios and 95% confidence intervals shown.

*Adjusted for iron deficiency, maternal education, socioeconomic status, mosquito net use, environmental risk, and maternity ward location.

**Table B in S2 Table. Multivariate linear regression results excluding children potentially exposed to lead by ingestion of fallen paint chips^§^.**

| Factor | Parasite density (mean logarithm)  (N=162)* |
| --- | --- |
| Blood lead level quartile |  |
| 1^st^ | 0 |
| 2^nd^ | -0.02 (-0.19, 0.14) |
| 3^rd^ | -0.02 (-0.20, 0.15) |
| 4^th^ | -0.01 (-0.18, 0.17) |

^§^ Coefficients and 95% confidence intervals shown.

*Adjusted for iron deficiency, maternal education, socioeconomic status, mosquito net use, environmental risk, malaria status before 12 months, and maternity ward location.
