# Supplementary material for: Blood lead level in infants and subsequent risk of malaria: A prospective cohort study in Benin, Sub-Saharan Africa
Source: PLoS One. 2019 Jul 18;14(7):e0220023. doi: 10.1371/journal.pone.0220023 (PMC6638975; doi:10.1371/journal.pone.0220023)
Supplement: S3 Table — (A) Multivariate negative binomial regression results for first 6 months after lead assessment. (B) Multivariate linear regression results for first 6 months after lead assessment. (DOCX) [file pone.0220023.s003.docx]

**Table A in S3 Table. Multivariate negative binomial regression results for first 6 months after lead assessment^§^.**

| Factor | Total malaria episodes (N=183)* | Total symptomatic episodes (N=183)* | Total asymptomatic episodes (N=183)* |
| --- | --- | --- | --- |
| Blood lead level quartile |  |  |  |
| 1^st^ | 1 | 1 | 1 |
| 2^nd^ | 0.96 (0.65, 1.42) | 1.17 (0.79, 1.73) | 0.64 (0.28, 1.46) |
| 3^rd^ | 0.87 (0.58, 1.31) | 1.02 (0.68, 1.54) | 0.62 (0.25, 1.51) |
| 4^th^ | 0.88 (0.58, 1.33) | 0.93 (0.61, 1.42) | 0.54 (0.22, 1.32) |

^§^ Incidence rate ratios and 95% confidence intervals shown.

*Adjusted for iron deficiency, maternal education, socioeconomic status, mosquito net use, environmental risk, and maternity ward location.

**Table B in S3 Table. Multivariate linear regression results for first 6 months after lead assessment^§^.**

| Factor | Parasite density (mean logarithm)  (N=183)* |
| --- | --- |
| Blood lead level quartile |  |
| 1^st^ | 0 |
| 2^nd^ | -0.06 (-0.25, 0.12) |
| 3^rd^ | -0.12 (-0.31, 0.08) |
| 4^th^ | -0.11 (-0.30, 0.08) |

^§^ Coefficients and 95% confidence intervals shown.

*Adjusted for iron deficiency, maternal education, socioeconomic status, mosquito net use, environmental risk, malaria status before 12 months, and maternity ward location.
