# Supplementary material for: Blood lead level in infants and subsequent risk of malaria: A prospective cohort study in Benin, Sub-Saharan Africa
Source: PLoS One. 2019 Jul 18;14(7):e0220023. doi: 10.1371/journal.pone.0220023 (PMC6638975; doi:10.1371/journal.pone.0220023)
Supplement: S1 File — (PDF) [file pone.0220023.s004.pdf]

ID number	Date of lead assessment	Blood lead level (µg/L)	Maternal education	SES quartile
1	4/14/2011	9.37	0	4
2	4/22/2011	20.7	1	3
3	5/11/2011	70	0	1
4	6/15/2011	41	0	2
5	5/2/2011	56	1	4
6	5/4/2011	4.81	1	3
7	5/13/2011	140	1	4
8	5/11/2011	15.9	1	3
9	5/10/2011	53	1	1
10	6/1/2011	85	1	2
11	5/20/2011	92	0	2
12	5/11/2011	58	1	3
13	5/20/2011	140	1	3
14	5/13/2011	71	0	1
15	5/23/2011	100	0	1
16	5/24/2011	40	0	2
17	5/24/2011	60	0	1
18	5/27/2011	35	1	4
19	6/17/2011	100	1	4
20	5/21/2011	44	0	1
21	6/14/2011	130	0	1
22	6/8/2011	82	0	4
23	6/7/2011	82	0	2
24	5/26/2011	75	0	2
25	5/15/2011	260	1	4
26	6/8/2011	80	0	1
27	6/6/2011	260	1	3
28	5/27/2011	30	0	2
29	6/10/2011	40	0	1
30	6/6/2011	22	0	2
31	6/6/2011	150	0	3
32	6/8/2011	52	1	3
33	6/10/2011	63	0	1
34	6/18/2011	32	0	4
35	6/2/2011	92	1	2
36	6/6/2011	54	0	2
37	6/15/2011	37	1	4
38	6/2/2011	91	1	4
39	6/14/2011	180	1	1
40	6/14/2011	140	0	1
41	6/15/2011	51	0	1
42	6/22/2011	42	1	1
43	6/15/2011	47	1	3
44	6/13/2011	140	0	4
45	6/23/2011	31	1	4
46	6/9/2011	150	1	1
47	6/8/2011	47	1	2
48	6/7/2011	39	0	3
49	6/27/2011	86	1	1
50	6/16/2011	41	0	2

51	6/8/2011	69	1	2
52	6/23/2011	19	1	1
53	6/27/2011	24	0	2
54	6/6/2011	63	0	3
55	6/8/2011	70	1	2
56	6/6/2011	30	1	3
57	6/15/2011	240	0	3
58	7/8/2011	110	1	1
59	6/23/2011	30	0	2
60	7/29/2011	54	0	1
61	6/30/2011	31	1	4
62	6/23/2011	76	1	1
63	7/13/2011	110	1	4
64	6/27/2011	37	0	1
65	6/22/2011	64	0	1
66	7/11/2011	87	0	2
67	7/5/2011	24	0	2
68	6/29/2011	54	1	4
69	7/8/2011	75	0	4
70	6/22/2011	110	1	2
71	7/5/2011	39	1	1
72	7/7/2011	35	1	4
73	7/1/2011	78	0	1
74	7/16/2011	85	1	4
75	7/1/2011	35	0	1
76	7/13/2011	53	1	1
77	7/5/2011	60		
78	7/7/2011	17	0	3
79	7/11/2011	33	1	2
80	7/18/2011	23	0	2
81	7/14/2011	73	1	3
82	8/18/2011	40	1	3
83	7/14/2011	50	1	4
84	8/3/2011	100	0	1
85	7/28/2011	92	0	4
86	7/18/2011	85	1	1
87	7/2/2011	58	0	1
88	8/4/2011	39	0	2
89	7/26/2011	65	1	2
90	8/4/2011	85	0	1
91	7/19/2011	35	1	4
92	7/18/2011	8.3	0	2
93	7/13/2011	57	0	1
94	7/20/2011	24	0	2
95	7/21/2011	36	0	1
96	7/21/2011	160	0	3
97	7/16/2011	29	0	2
98	7/25/2011	39	1	2
99	7/29/2011	35	1	3
100	7/27/2011	51	0	3
101	7/21/2011	63	0	2
102	8/1/2011	72	1	2

103	8/1/2011	110	0	3
104	7/28/2011	60	0	4
105	8/1/2011	78	0	2
106	7/29/2011	39	0	1
107	8/1/2011	32	0	1
108	7/25/2011	55		
109	8/24/2011	230	1	2
110	7/30/2011	38	0	3
111	9/8/2011	33	0	1
112	8/4/2011	63	0	1
113	8/9/2011	97	0	4
114	8/9/2011	52	1	1
115	7/30/2011	43	1	4
116	7/27/2011	20	0	1
117	8/4/2011	52	0	4
118	8/10/2011	71	1	2
119	7/27/2011	39	0	2
120	8/7/2011	64	1	4
121	8/9/2011	81	1	4
122	7/27/2011	57	0	2
123	7/27/2011	130	0	1
124	8/4/2011	49	1	4
125	9/2/2011	100	1	2
126	8/14/2011	54	0	2
127	8/10/2011	31	0	2
128	8/10/2011	33	1	3
129	8/16/2011	48.5	1	4
130	8/17/2011	120	0	3
131	8/26/2011	87	0	2
132	8/29/2011	110	0	1
133	8/10/2011	53	0	4
134	8/9/2011	88	0	1
135	8/23/2011	56	0	2
136	8/29/2011	51	0	1
137	8/22/2011	16	1	4
138	9/5/2011	70	0	4
139	8/23/2011	56	1	2
140	8/26/2011	93	0	1
141	8/22/2011	64	1	2
142	8/26/2011	95	0	3
143	9/9/2011	46.4	0	3
144	8/31/2011	34	1	4
145	8/25/2011	46	1	1
146	8/26/2011	99	0	1
147	8/12/2011	57	0	1
148	8/29/2011	100	0	2
149	9/7/2011	36	0	1
150	8/23/2011	59	0	1
151	8/31/2011	61	1	4
152	9/2/2011	23	0	1
153	9/2/2011	66	1	3
154	9/19/2011	50.1	1	4

155	9/7/2011	40	1	4
156	8/29/2011	110	0	2
157	10/13/2011	49.1	1	1
158	11/17/2011	37.9	0	2
159	10/31/2011	41	1	2
160	11/25/2011	28.6	0	2
161	12/5/2011	76.3	1	2
162	12/21/2011	88.5	0	1
163	12/21/2011	66.1	0	1
164	11/30/2011	578	1	4
165	12/19/2011	60.9	1	3
166	1/18/2012	51.6	1	3
167	1/5/2012	47.5		
168	1/5/2012	88.5	0	3
169	1/3/2012	105	0	4
170	1/25/2012	59.7	0	2
171	1/11/2012	34	1	4
172	1/11/2012	42.5	0	4
173	1/23/2012	176	0	2
174	1/25/2012	114	0	2
175	2/6/2012	33.2	0	1
176	2/2/2012	72.7	1	4
177	2/27/2012	102	0	1
178	2/15/2012	21.1	0	2
179	3/3/2012	43.1	0	
180	3/7/2012	40	0	4
181	3/16/2012	29	0	1
182	3/20/2012	49.9	0	2
183	5/7/2012	117	1	3
184	3/6/2012	30.3	1	3
185	3/28/2012	53.7	0	2
186	4/4/2012	253	1	2
187	4/19/2012	86.4	0	1
188	4/17/2012	82.1	0	2
189	5/10/2012	168	0	2
190	4/24/2012	21.3	1	2
191	5/4/2012	39.4	1	3
192	5/21/2012	36.1	0	3
193	5/18/2012	73.4	0	1
194	5/14/2012	136	1	3
195	5/9/2012	66.1	1	3
196	5/8/2012	33.6	1	4
197	5/10/2012	49.5	1	2
198	5/11/2012	76.3	0	1
199	5/23/2012	57.2	1	4
200	5/18/2012	50.6	0	2
201	6/26/2012	62	0	2
202	7/6/2012	105	0	2
203	5/23/2012	52.4	1	4
204	7/4/2012	44.8	0	2
