# Supplementary material for: Blood lead level in infants and subsequent risk of malaria: A prospective cohort study in Benin, Sub-Saharan Africa
Source: PLoS One. 2019 Jul 18;14(7):e0220023. doi: 10.1371/journal.pone.0220023 (PMC6638975; doi:10.1371/journal.pone.0220023)
Supplement: S2 File — (PDF) [file pone.0220023.s005.pdf]

ID number	Total malaria episodes	Total symptomatic episodes	Total asymptomatic episodes	Mosquito net use frequency	Mean log environmenal risk
1	5	4	1	4	-0.001220131
2	2	2	0	4	0.107836708
3	7	6	0	4	0.002544084
4	3	3	0	4	-0.166105285
5	0	0	0		
6	2	0	2	4	0.258777499
7	2	1	0	4	0.129636943
8	1	1	0	4	0.001694759
9	14	5	9	4	0.040215466
10	1	1	0	4	-0.287600756
11	3	3	0	3	-0.100969523
12	0	0	0		-0.039836679
13	0	0	0		0.083427034
14	0	0	0		0.057249106
15	1	1	0	3	0.125431076
16	3	2	1	4	-0.16305615
17	0	0	0		0.242938846
18	0	0	0	4	-0.045085441
19	2	2	0	4	0.081231073
20	6	4	2	3	0.314816177
21	1	1	0	4	-0.205918029
22	3	3	0	4	0.130674899
23	3	3	0	2	-0.158419758
24	1	1	0	3	0.173944905
25	2	2	0	4	0.021585071
26	3	2	1	2	-0.058909226
27	0	0	0	4	0.107736781
28	2	1	0	3	-0.157483503
29	4	4	0	4	-0.042506985
30	5	3	2	1	-0.172034815
31	2	1	0	4	0.209546253
32	3	3	0	4	0.15230836
33	4	3	1	2	-0.144048676
34	2	2	0	4	0.151934877
35	3	3	0	4	0.034118783
36	2	1	1	4	-0.214559346
37	2	2	0	3	0.089067064
38	6	3	3	4	0.18048656
39	6	5	1	4	0.070105597
40	0	0	0	4	0.107111476
41	3	2	1	4	-0.00805884
42	2	2	0	4	-0.079980351
43	0	0	0	4	0.277536392
44	3	1	2	4	-0.143572107
45	4	4	0	4	0.287826836
46	0	0	0		0.137338161
47	1	1	0	4	0.046662327
48	1	1	0	4	0.228029788
49	1	0	1	4	-0.034425493
50	2	0	2	4	0.055249855
51	4	4	0	4	0.163708761

52	6	2	4	4	0.363206655
53	5	4	1	4	-0.002022432
54	4	2	2	4	0.199235141
55	3	2	1	4	-0.084936745
56	3	1	2	4	0.140315235
57	7	5	2	4	0.160997719
58	2	2	0	4	0.282788068
59	4	1	3	4	-0.062405564
60	0	0	0		0.178524286
61	1	0	0	4	0.130864888
62	1	1	0	4	-0.307386428
63	1	0	1	4	0.149981424
64	2	1	1	4	-0.145373747
65	0	0	0	4	-0.220782474
66	1	1	0	3	-0.043503445
67	2	0	2	4	-0.339258105
68	0	0	0	4	0.140985712
69	3	2	1	4	0.249068484
70	1	1	0	4	-0.049451176
71	6	0	6	4	0.08151491
72	0	0	0	4	0.030812079
73	2	2	0	4	0.134854659
74	5	5	0	2	0.1192609
75	1	1	0	4	-0.070138462
76	5	4	1	3	0.167181164
77	2	2	0	4	0.047208246
78	1	1	0	4	-0.06331221
79	2	2	0	3	-0.036136203
80	7	2	5	4	0.267260551
81	2	2	0	3	0.125188857
82	1	1	0	4	0.289594322
83	4	3	0	4	0.262229234
84	0	0	0		
85	2	1	0	4	0.016646314
86	8	2	6	3	-0.075986966
87	1	1	0	4	0.277929664
88	3	2	0	4	-0.049507644
89	5	2	3	4	0.032060452
90	0	0	0	4	-0.193832457
91	4	3	1	4	-0.032299966
92	9	1	7	4	0.120200545
93	5	4	1	3	-0.028203273
94	1	1	0		0.338073224
95	8	6	1	4	0.340774953
96	2	2	0	4	0.168866053
97	10	7	3	4	0.28794387
98	0	0	0	4	-0.193090186
99	13	5	7	1	0.219758198
100	8	5	2	4	0.371635467
101	3	3	0	4	0.424509019
102	5	3	2	4	0.089105412
103	2	1	0	4	0.29295513
104	10	8	2	2	0.091698356
105	2	2	0	2	-0.154774785

106	3	3	0	4	0.299731672
107	1	1	0	4	-0.258067369
108	1	1	0	3	0.055259917
109	4	2	2	4	-0.020212105
110	1	1	0	3	-0.186065987
111	3	3	0	2	-0.165029123
112	6	5	1	4	0.027467474
113	1	1	0	4	0.056864932
114	2	2	0	3	-0.278472155
115	1	1	0	4	0.183237866
116	2	2	0		-0.097757638
117	0	0	0	4	-0.014774962
118	0	0	0	4	-0.061577696
119	11	7	4	4	-0.022516092
120	7	4	3	4	0.135432571
121	0	0	0	4	0.025054611
122	1	1	0	4	0.105307236
123	8	2	5	4	-0.065250166
124	1	1	0	4	0.421613514
125	3	3	0	4	0.224779114
126	7	6	1	4	-0.04891666
127	5	3	2	2	-0.178105921
128	3	3	0	4	0.353949666
129	5	4	1	4	0.29692632
130	3	3	0	4	-0.012754435
131	3	1	2	4	0.217001915
132	6	5	0	4	-0.160212383
133	2	1	1	4	-0.011351177
134	1	0	1	3	-0.25082022
135	2	2	0	2	-0.160720319
136	5	3	2	4	0.176325977
137	1	0	1	4	0.083879769
138	8	4	4	4	0.051328462
139	1	1	0	4	0.001678747
140	2	2	0	4	0.153798327
141	3	2	1	3	-0.150685802
142	0	0	0		
143	3	3	0	4	0.105377868
144	0	0	0	4	0.103257559
145	1	1	0	4	-0.024645798
146	4	3	1	4	0.21934174
147	1	1	0	4	-0.075162493
148	1	1	0	3	-0.110026516
149	2	1	0	4	-0.129253909
150	2	1	1	4	0.005564278
151	5	5	0	4	0.228282303
152	1	1	0	2	-0.187630355
153	4	0	4	4	0.093413621
154	2	2	0	4	0.158496454
155	2	1	1	4	0.017836478
156	2	2	0	1	-0.168360889
157	0	0	0		
158	2	2	0	2	-0.157136366
159	3	3	0	1	-0.214269832

160	7	7	0	4	0.051560201
161	1	1	0	4	0.07906311
162	1	1	0	2	-0.030776961
163	2	1	1	3	-0.021590341
164	1	1	0	4	0.127218962
165	0	0	0		0.135646597
166	1	1	0	2	0.53467232
167	1	1	0	3	-0.122158229
168	5	5	0	4	-0.013840498
169	0	0	0		0.190450773
170	2	2	0	4	-0.233424425
171	2	2	0	4	0.151594132
172	0	0	0		0.101058871
173	1	1	0	4	-0.037495248
174	5	2	2	4	-0.102123007
175	1	1	0	4	-0.059753541
176	4	3	1	4	0.179242611
177	2	2	0	2	-0.193191007
178	6	5	0	3	0.127673566
179	0	0	0	4	-0.243188977
180	5	5	0	4	0.046981059
181	3	3	0	4	-0.024732186
182	8	5	2	4	0.217282817
183	8	5	2	4	0.131248176
184	0	0	0	4	0.224063948
185	4	3	1	2	-0.31429261
186	6	2	3	4	-0.033015106
187	7	4	3	3	-0.160151437
188	3	2	1	4	0.038124789
189	3	2	1	4	-0.02004214
190	0	0	0	4	0.020298308
191	1	1	0	4	-0.068283267
192	3	3	0	4	-0.234131381
193	8	6	2	4	-0.026315939
194	0	0	0	4	0.008345521
195	2	2	0	3	0.182715341
196	5	4	1	4	0.108953334
197	0	0	0	4	-0.18317695
198	2	2	0	4	0.181786716
199	4	3	1	4	0.135596246
200	0	0	0	4	-0.114709713
201	3	3	0	4	-0.061205823
202	2	2	0	4	-0.017670395
203	4	2	2	4	0.0281976
204	3	3	0	3	-0.125461191
